# Salol

**Published:** 1895-08

**Authors:** R. M. Sanger

**Affiliations:** Orange, N. J.


### SALOL.[1]

BY R. M. SANGER, D.D.S., ORANGE, N. J.

Mr. President and Gentlemen,—If originality of thought or newness in treatment are prerequisites in writing a paper that shall be profitable, there can be no excuse for this paper. But if observation and experience have any value, I can reasonably expect to make the subject chosen one of interest to you.

In the *Dental Cosmos* for May, 1894, there appeared an able article by Dr. A. E. Mascott, of Paris, France, on "Salol in Root-Filling," which impressed me to such a degree that I at once began experimenting with this material. The result was most gratifying, and salol has become a valuable addition to my list of remedies.

Dr. Mascott describes the drug and its action as follows: "Salol is a compound in which one hydrogen atom of the salicylic acid has been replaced by the radical phenyl. To obtain it the perchloride of phosphorus or the oxychloride of carbon, or nascent chlorydic acid, is made to act on a mixture of salicylate and phenate of sodium. It is also obtained by another method. At 120° C. the oxychloride of phosphorus is made to act on a mixture of salicylic acid and phenol, producing the salicylate of phenyl, or salol. Thus obtained, it is in the form of a white, crystalline powder, insoluble in water and glycerin, but readily soluble in ether, alcohol, chloroform, benzine, spirit of turpentine, copal balsam,

[1] Read before the Odontological Society of Pennsylvania, February 9, 1895.

liquid vaseline, fixed and volatile oils. It has a fragrant taste, and smells similar to the essence of winter-green. At 40° C. it completely fuses into a liquid. When the temperature is raised to or near its boiling-point, it retains the liquid state after the temperature has again fallen below its melting-point. Even at the ordinary temperature of the office-rooms it will remain fluid from fifteen to twenty minutes; but if fused at the lowest possible heat it then returns in a few minutes to the solid state. This substance has also the peculiar property of forming intimate mixtures with iodoform, aristol, and camphor. Melted together, salol and aristol, salol and iodoform, become liquid like salol alone, which form in a few minutes a solid mass, not a new chemical product, but a mixture of two substances, having each one of them all of its characteristic properties, excepting the new form of crystallization. Salol was proposed by Professor Nencki as a substitute for salicylate of sodium. Its general use is that of an antiseptic."

As a root-filler, salol is manipulated in this manner: Having the rubber dam adjusted, the cavity dry, and the canals sterilized by any of the various methods at our command, the root is thoroughly dried with an Evans root-drier, and the salol crystal placed in the cavity with a flat spatula or an amalgam carrier. The hot root-drier is then passed through the powder, which immediately melts and follows the instrument into the canal, following by capillary attraction beyond the point where the instrument stops. Repeating this once or twice, the canal will be entirely filled with the liquid salol. A warm tin or copper canal-point is then introduced, and carried carefully as far into the canal as possible without passing through the apex. It is then left for a few moments to cool, when the canal-point will be found to be solidly embedded in a crystalline, antiseptic mass which entirely fills the canal. If the cavity is so situated that the crystals cannot be readily placed in position, the salol is first melted in a test-tube, care being taken not to heat it more than just enough to liquefy the powder. Then by the use of a glass syringe, slightly warmed, the salol is placed in the cavity, and carried to the end of the canal by the use of the root-drier as before.

I have yet to record a single failure from this use of this material, and I believe it to be as near the ideal root-filling as anything yet offered to the profession.

If from any cause we should desire to remove the filling, a warm instrument held against the metal point will quickly heat it through, causing the salol to melt, and the whole can be readily washed out of the cavity with alcohol without any undue pressure or irritation to the sore tooth.

Just a word here regarding these little glass syringes. They are made of blown glass, and may be heated over an alcohol or Bunsen flame, and the point bent to any desired shape without fear of breaking if care be used when the heat is first applied. They are very cheap, costing about one dollar per dozen, and will be found extremely useful where a syringe is needed.

We frequently have cases presented where the teeth have decayed to such an extent that to remove all the infected tissue would expose the pulp, and to leave it would almost certainly insure exposure and trouble from recurrent decay unless thorough sterilization can be secured and maintained. Let us suppose the case of an inferior first permanent molar. Having adjusted the dam, dried the cavity, and removed as much of the infected material as the condition will admit, sterilizing the remainder as thoroughly as possible, by the use of whatever non-irritating germicide the operator may prefer, the cavity is then thoroughly dried, and the crystals of salol placed in with a flat spatula, and melted by passing over it a smooth, flat burnisher or amalgam plugger warmed sufficiently to liquefy the salol without causing pain to the patient. The less heat used the more quickly crystallization takes place again, which should occur in three or four minutes at the longest. We have now sealed the floor of the cavity with a firm mass of antiseptic material capable of receiving considerable pressure without inconvenience to the patient. This is far from being a non-conductor, however, and should be covered with a layer of cement before the final filling is inserted.

In treating an upper tooth under similar circumstances the only difference would be in the manner of introducing the salol. Here we take a piece of asbestos felt, cut to fit the floor of the cavity, warm it over a spirit lamp, and place it against the salol crystals, which have been placed on a glass slab for that purpose. Immediately the salol is melted and taken up by the asbestos, until it is more than saturated. It is then placed in the cavity, and, upon cooling, we proceed as before, not forgetting the cement, however, as the asbestos thus treated does not give a non-conducting surface.

Where the pulp is actually exposed, and we desire to cap it, remove as much decay as possible around the edge of the exposure, and saturate the floor of the cavity with oil of cloves or cinnamon to relieve the pain, if any be present, and sterilize the cavity. A small disk of platinum, about 32 gauge, is then cut to fit the floor of the cavity, and the centre depressed with a ball burnisher, thus forming a metal cup. A paste is made of carbolic acid, oxide of zinc, and vaseline, with which the cup is filled, and placed gently over the exposed pulp. The crystals of salol are then placed over the cap and liquefied by passing over them a warm, flat burnisher. In a few moments the cap and floor of the cavity are hermetically sealed, and the tooth is ready for filling.

Salol is readily dissolved by any of the volatile oils or alcohol. All surplus should be carefully removed from around the edges of the cavity and the outside of the tooth, as it is irritating to the mucous membrane, and will make an abrasion somewhat resembling a canker if left in contact with the gum. This sore is not dangerous, however, and will heal in a day or two, the healing being hastened by the application of vaseline.

